# Emotion Talk in Psychotherapy: A Systematic Review of Conversation Analytic Studies

**DOI:** 10.1002/cpp.70297

**Published:** 2026-06-16

**Authors:** Shuai Zhang, Tiantian Huang, Haiying Li, Lu Chen

**Affiliations:** ^1^ School of Foreign Languages University of Jinan Jinan Shandong China; ^2^ School of Humanities Ludong University Yantai China; ^3^ Health Studies Research, Methodologies, Florence Nightingale Faculty of Nursing, Midwifery & Palliative Care King's College London London UK

**Keywords:** conversation analysis, emotion talk, psychotherapy, systematic review, therapist–client interaction

## Abstract

Emotional communication is central to psychotherapy and plays a key role in shaping clients' experiences and therapeutic change. Conversation analysis (CA) offers a process‐oriented approach to examining how emotions are displayed, managed, and responded to in moment‐by‐moment therapeutic interaction. Although a growing line of CA research has focused on emotion talk in psychotherapy, an integrative synthesis of this literature remains limited. This review synthesizes CA studies on emotion talk in psychotherapy, with a focus on identifying key interactional processes through which emotion talk is produced and managed in naturally occurring therapeutic encounters. Following PRISMA guidelines, we searched six major databases and identified 18 studies that met the inclusion criteria. The thematic analysis revealed two overarching themes: (1) the sequential organization of emotion talk, including clients' emotional displays, therapists' emotional management, and clients' responses; and (2) changes in therapeutic interaction, encompassing shifts in clients' emotional experiences and the therapeutic relationship over time. By integrating CA studies on emotion talk in psychotherapy, the review clarifies the interactional resources through which therapists and clients navigate emotional moments. These findings advance understanding of psychotherapy as an interactional achievement and offer practice‐relevant insights into how therapists can attend to and work with clients' emotions to support further therapeutic work.

## Summary

- Therapists can benefit from attending to how emotion emerges sequentially in clients' talk, rather than treating emotion only as a separately reportable inner state.
- Clients' emotional displays may be conveyed through verbal, prosodic, and embodied resources; attending to these resources can help therapists recognize opportunities for emotional exploration.
- Therapists' responses to clients' emotional displays can shape subsequent interaction, including whether emotion talk is sustained, redirected, resisted, or further elaborated.
- The review highlights interactional practices through which clients display emotions and therapists respond to these emotional displays, informing therapists' reflective practice and supporting more responsive emotional communication in psychotherapy.

## 1 Introduction

Emotions are usually conceptualized as a psychological experience characterized by high intensity and strong emotional value (either pleasure or unpleasantness) (Cabanac 2002). It is increasingly recognized that emotions are generated by complex interactions between biological, psychological, cultural, and social processes (Voutilainen et al. 2010a). Emotion regulation refers to the process of what emotions an individual has, when these emotions are generated, and how they experience or express these emotions (Gross 1998). It encompasses five processes: situation selection, situation modification, attentional deployment, cognitive change, and response modulation (Gross and Thompson 2007). Emotion regulation can be intrinsic, targeting one's own emotional experience, or extrinsic, targeting others' emotions, as seen when parents cope with their children's emotions (Gross and Jazaieri 2014; Macklem 2008). Given that difficulties in either emotion generation or emotion regulation are closely associated with emotional and affective problems, emotion regulation is considered a key factor in addressing psychological disorders and enhancing overall well‐being (Gross and Ford 2024; Kraiss et al. 2020; Visted et al. 2018). Over one billion individuals globally are experiencing mental health disorders, a substantial portion of which involve emotional regulation dysfunction, which seriously undermines the quality of life of individuals and puts a heavy burden on the social, economic, and public health systems (World Health Organization 2025). In this context, psychotherapy plays a crucial role in treating emotional disorders. It enables clients to understand, regulate, and transform their emotional experiences and associated behaviours (Antaki 2008; Peräkylä et al. 2008a; Peräkylä 2019).

In psychotherapy, emotions occupy a central role in both comprehending and facilitating particular types of therapeutic change (Greenberg and Safran 1989). In psychotherapy traditions, emotional change has been treated as central to therapeutic transformation, including changes in cognition and behaviours (Greenberg 2015). In this line of work, helping clients identify, experience, accept, and explore emotion is understood as enabling greater access to important information about themselves and the world, thereby supporting a more vital and adaptive way of living (Greenberg 2002). Some approaches to psychotherapy recognize the importance of emotion; however, they differ in how emotion is understood and addressed in practice. Classical psychoanalysis and behaviour therapy have offered different ways of conceptualizing emotion in psychotherapy: the former typically views emotions as manifestations of unconscious instinctual drives, whereas the latter emphasizes that human behaviors are controlled by learned stimulus–response associations (Greenberg et al. 1993). Client‐centred therapy integrates emotion into the experiential context, highlighting the unity of emotion and cognition in the present moment (Rogers 1959). Process‐experiential therapy conceptualizes emotion as organizing processes that support adaptive functioning and problem‐solving, while emphasizing the importance of increasing clients' awareness of emotion so that it can function as orienting information for navigating their environment (Greenberg et al. 1993; Greenberg and Safran 1989).

Conversation analysis (CA), rooted in ethnomethodology, is a distinctive approach to the study of social interaction (Sacks 1972). As an inductive approach, CA draws on naturally occurring talk as its primary data and examines interactional practices to reveal how social interaction is accomplished through the sequential organization of talk‐in‐interaction (Heritage and Maynard 2006; Schegloff 2007). It describes how participants produce and jointly organize social actions through the unfolding of turns at talk, while revealing the underlying rules, meanings, and structures that sustain interactional order (Heritage 1984; Sacks et al. 1974). Building on this foundation, CA treats emotion not as a private inner state but as a social phenomenon, attending to its sequential organization and its functional relationship with social action (Robles and Weatherall 2021). In contrast to traditional approaches that rely primarily on scales, interviews, or post hoc reports, CA offers empirical tools for examining how emotion is displayed, managed, and made consequential in naturally occurring interaction (Hepburn and Potter 2023). Specifically, CA analyzes how emotional displays, affective stances, and action formation are systematically organized in relation to participants' ongoing interactional projects (Peräkylä and Sorjonen 2012). This analytic perspective is especially relevant to psychotherapy, where clients' emotional displays and therapists' responses are central to the accomplishment of the therapeutic work.

Emotion is inherently tied to the embodied manifestation of participants' stance, publicly displayed and interactionally accomplished in talk‐in‐interaction through a range of multimodal resources, including language, posture, and other symbolic forms (Du Bois 2007; Goodwin et al. 2012; Stivers and Sidnell 2005). Emotional displays are therefore rarely accomplished through words alone; instead, they emerge through the multimodal organization of action (Ruusuvuori 2012; Sidnell and Stivers 2012). Furthermore, these multimodal resources are coordinated sequentially, allowing participants to display, recognize, and respond to emotion in ways that negotiate meaning and shape the trajectory of the ongoing interaction (Knapp and Daly 2011; Mondada 2006; Ruusuvuori and Peräkylä 2009). For instance, facial expression may persist beyond the completion of a verbal turn, thereby sustaining the affective orientation displayed in prior talk (Mondada 2006; Ruusuvuori and Peräkylä 2009). By examining how therapists and clients display and respond to emotion in real time through these multimodal resources, CA provides a rigorous method for understanding how emotional experience is co‐constructed, how the therapeutic relationship is built and maintained, and how psychotherapeutic change is accomplished in and through interaction (Peräkylä 2019; Wilkinson and Kitzinger 2006). A review that focuses on CA research on emotion in psychotherapy is therefore needed to synthesize how emotion becomes observable and actionable in the fine details of therapeutic interaction.

Emotional communication between therapists and patients is central to the psychotherapy process and outcome (Ha and Longnecker 2010; Roter et al. 2006). Although specific treatment techniques account for some variation in outcome, the quality of the therapeutic relationship has been shown to exert a stronger influence on therapeutic effectiveness (Norcross 2011). At the core of this relationship lies the therapeutic alliance, which entails the establishment of a mutual bond and the reciprocal feelings between participants (Bordin 1979). In this context, therapists' responses to clients' emotional disclosures are particularly important. Positive responses can improve the effect of treatment (Cape et al. 2000; Jani et al. 2012) and enhance the patient's satisfaction and self‐efficacy (Finset 2010; Zachariae et al. 2003). Conversely, misalignment in emotional communication may lead to misunderstanding, resistance and even the breakdown of the therapeutic relationship (Voutilainen et al. 2010b). Broader psychotherapy research has contributed to the understanding of the importance of emotional communication for therapeutic outcome at a relatively general level, for example, by emphasizing the importance of empathy, alliance, or emotion regulation as treatment factors.

The existing reviews have established the broader significance of emotion for psychological well‐being and therapeutic effectiveness, examining emotion in psychotherapy from various perspectives, including emotion regulation, treatment processes and therapy outcomes. For instance, Niu et al. (2025) have concentrated on psychotherapy outcomes, investigating the impact of communicative behaviours (e.g., empathy, shared decision‐making, and emotional support) on treatment outcomes. Other reviews have emphasized emotion regulation in psychotherapy, identifying emotion regulation difficulties associated with various mental disorders (Lincoln et al. 2022; Sheppes et al. 2015) and exploring interventions targeting emotion dysregulation (Saccaro et al. 2024). On this basis, CA research further advances understanding by showing how the emotion talk is constructed in real time through participants' turn‐by‐turn and multimodal behaviours. More relevant to the present review is Peräkylä's (2019) review, synthesizing CA studies in psychotherapy, which concentrates on the sequential organization of interaction and elucidates how therapeutic change emerges in and through talk‐in‐interaction. Building on this work, this review narrows the analytical focus specifically to emotion talk in psychotherapy, offering a more fine‐grained account of the interactional practices. To the best of our knowledge, there is no systematic review of CA studies on emotion talk in psychotherapy. Therefore, this review aims to comprehensively analyse the latest CA research on emotion talk in psychotherapy, clarify the interactional sequence and dynamic process behind emotion talk, and provide insights to promote the progress of treatment.

## 2 Methods

This study employed a systematic review as its research method, aiming to examine the latest research on emotion talk in psychotherapy. The included studies varied widely in countries, treatment practices, research designs, and outcome measures. All included studies employed qualitative methodologies. It is due to the heterogeneity of the outcome variables that a quantitative synthesis through meta‐analysis was not feasible. To ensure the objectivity of the research process, we followed the PRISMA guidelines for a systematic review.

### 2.1 Search Strategy

We carried out a comprehensive literature search in six high‐quality electronic databases between October 2024 and November 2024, which covered fields such as medicine, sociology, and psychology. These databases included Web of Science, PubMed, ScienceDirect, CNKI, ProQuest, and Embase. No restriction was placed on the starting year of publication; the search covered all records available in each database up to November 2024, with an updated search conducted in April 2026. To maximize the sensitivity of the search, we have adopted a search strategy that combines free text keywords and MeSH words, with the core search terms focusing on “emotion” and “conversation analysis”. See Table 1 for the detailed search strategy. We limited the search scope to peer‐reviewed articles in English or Chinese. In order to avoid missing relevant studies, we also manually screened the lists of references in the included articles. All retrieved literature records were imported into the Covidence literature management system for screening.

**TABLE 1:** Search keywords.

**#1** (affect OR anger OR anxiety OR apathy OR boredom OR courage OR depression OR disgust OR euphoria OR emotion OR fear OR feeling OR feelings OR forgiveness OR frustration OR grief OR guilt OR happiness OR hate OR joy OR jealousy OR loneliness OR love OR mind OR mood OR passion OR panic OR pleasure OR rage OR regret OR sadness OR shame)
**#2** (adjacency pair OR backchannel OR CA OR closing section OR conversation analyses OR conversation analysis OR conversation analysis approach OR conversation analysis based* OR conversation analysis literature OR conversation analysis method* OR conversation analysis perspective OR conversation analysis technique* OR conversation analysis tool* OR conversation analysis* OR conversation analytic* OR conversation analytic approach OR conversation analytic findings OR conversation analytic method OR conversation analytic perspective OR conversation analytic research OR conversation analytic stud* OR conversational turn‐taking OR formulation OR opening section OR repair mechanism OR sequence organization OR turn construction unit OR turn transition OR TCU)
**#1 AND #2**

### 2.2 Eligibility Criteria

This review aimed to systematically integrate the research on emotion talk in psychotherapy based on the CA framework. We followed the Population, Intervention, Comparison, and Outcome (PICO) framework and formulated the following inclusion criteria: (1) clients with emotional disorders; (2) research in psychotherapy; (3) using CA methods; (4) research focusing on communication in emotion talk. The following studies were excluded: (1) clients did not have emotional disorders; (2) the study was not conducted in psychotherapy; (3) CA methods were not used; (4) it was not related to communication in emotion talk. In addition, if the research is not peer‐reviewed, non‐journal publications, or written in languages other than English and Chinese, it will also be excluded. The reason for setting language restrictions is that all members of the research team are proficient in Chinese and English, so as to ensure the accuracy and reliability of the research screening.

### 2.3 Study Selection

**FIGURE 1:**
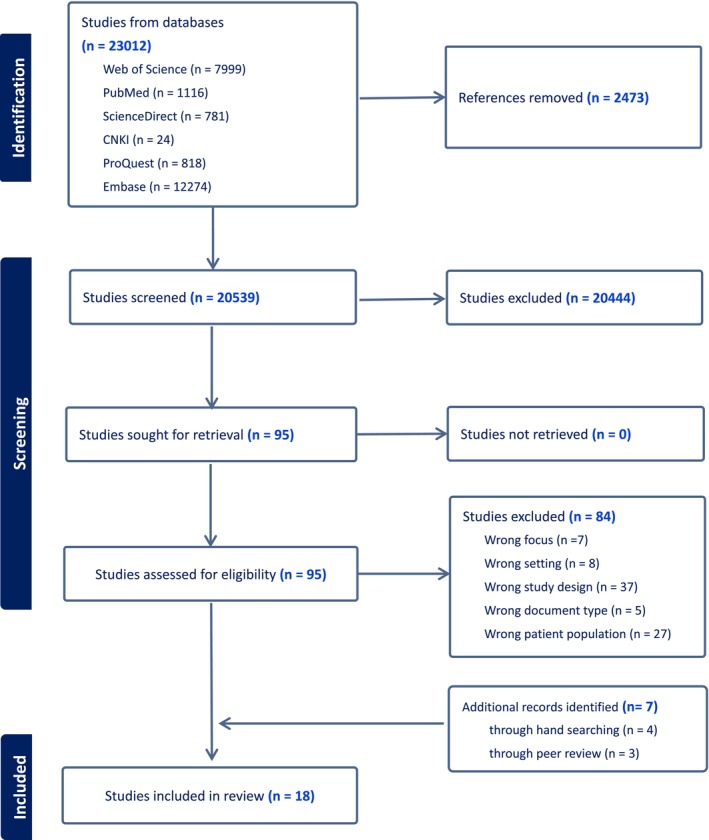
Flow chart of the literature selection.

Through database search, a total of 23,012 records were initially identified. We used Covidence software to manage the screening and review process. The software automatically removed duplicate records, with a total of 2473 duplicate records. The remaining 20,539 records were screened by the title and summary. At this stage, predefined inclusion and exclusion criteria were applied to screen these studies. Records that were clearly outside the scope of the review were excluded; for example, the study was not related to psychotherapy, not concerned with psychotherapy or therapeutic encounters, or not focused on emotion or communication, resulting in 95 studies retained for further screening. Subsequently, the full texts of the remaining 95 studies were evaluated to assess eligibility in detail. The inclusion criteria were as follows: (1) studies involving clients with emotional disorders; (2) studies conducted in psychotherapy settings; (3) studies employing CA; and (4) studies focusing on emotional communication or emotion talk. Based on these criteria, 84 studies were excluded. All screening procedures were carried out independently by two reviewers, and any disagreements were resolved through discussion and, if necessary, consultation with a third reviewer to reach a consensus. Ultimately, 11 studies that met the inclusion criteria were included. In addition, 7 studies were identified through manual retrieval of references, and a total of 18 studies were included in the final comprehensive analysis. The screening process is summarized in the PRISMA flowchart (Figure 1).

### 2.4 Data Extraction

We extracted relevant data characteristics for each included article to analyse the study content. The key features of the collection included: author(s), year of publication, countries, journal, language of data, data collection methods, setting, and research design. Two reviewers independently collected the data from each study, with any discrepancies resolved through discussion with a third reviewer. See Table 2 for details.

**TABLE 2:** Study characteristics of the 18 included studies.

Author(s) and year	Journal	Country	Language of data	Data collection method	Setting	Research design
Peräkylä (2005)	Communication & Medicine	Finland	Finnish (translated into English)	Audio	Therapist: 2 psychoanalytic analysts Client: 3 patients Type of therapy: psychoanalytic psychotherapy	Qualitative
Voutilainen et al. (2010a)	Research on Language and Social Interaction	Finland	Finnish (translated into English)	Audio	Therapist:1 Finnish‐speaking therapist Client: 1 patient Type of therapy: cognitive‐constructivist therapy	Qualitative
Voutilainen et al. (2010b)	Qualitative Research in Psychology	Finland	English	Audio	Therapist: 1 therapist Client: 1 patient with depression Type of therapy: cognitive‐constructivist therapy	Qualitative
Voutilainen et al. (2011)	Psychotherapy Research	Finland	English	Audio	Therapist: 1 female cognitive therapist Client: 1 20‐year‐old female patient with anxiety and depression Type of therapy: cognitive‐constructivist therapy	Qualitative
Muntigl et al. (2013)	Journal of Pragmatics	Canada	English	Video	Therapist: female emotion‐focused therapists Client: female clients with clinical depression Type of therapy: emotion‐focused therapy	Qualitative
Muntigl et al. (2014)	Discourse Studies	Canada	English	Video	Therapist: emotion‐focused therapists Client: clients with clinical depression Type of therapy: emotion‐focused therapy	Qualitative
Muntigl and Horvath (2014a)	Research on Language and Social Interaction	Canada	English	Video	Therapist: four therapists (1 emotion‐focused therapist, 1 Gestalt therapist, 1 narrative therapist and 1 symbolic experiential therapist) Client: six clients Type of therapy: 4 experiential therapies (Gestalt therapy, emotion‐focused therapy, narrative therapy and symbolic experiential therapy)	Qualitative
Muntigl and Horvath (2014b)	Psychotherapy Research	Canada	English	Video	Therapist: two female emotion‐focused process experiential therapists Clients: two female clients with depression Type of therapy: emotion‐focused therapy	Qualitative
Sutherland et al. (2014)	Psychotherapy Research	UK	English	Audio, Video	Therapists: 3 therapists Client: seven clients (one male, six female) Type of therapy: emotion‐focused therapy	Qualitative
Weiste and Peräkylä (2014)	Psychotherapy Research	Finland	English	Audio, Video	Therapist: two therapists (1 cognitive therapist and 1 psychoanalyst) Client: four clients Type of therapy: 2 therapies (psychoanalysis and cognitive therapy)	Qualitative
Muntigl et al. (2019)	Psychotherapy Research	Canada	English	Video	Therapist: 3 therapists (1 psychoanalyst, 1 emotion‐focused therapist and 1 client‐centred therapist) Client: 3 female clients with depression Type of therapy: 2 therapies (emotion‐focused therapy and client‐centred therapy)	Qualitative
Deppermann et al. (2020)	Frontiers in Psychology	Germany	German (translated into English)	Video	Therapist: 2 therapists (1 young female therapist in analytic training and 1 psychoanalytically trained senior therapist) Client: 2 clients (1 male client in his late 60s with depression and a functional pain syndrome, and 1 young woman in her 20s with psychogenic seizures) Type of therapy: psychodynamic therapy	Qualitative
Muntigl (2020)	Frontiers in Psychology	Canada	English	Video	Therapist: 1 female client‐centred therapist Client: 1 female client with depression Type of therapy: client‐centred therapy	Qualitative
Guxholli et al. (2021)	Frontiers in Psychology	Albania	Albanian (translated into English)	Video	Therapist: two female therapists Client: two clients (1 female, 1 male) Type of therapy: psychoanalytic therapy	Qualitative
Ma et al. (2022)	Frontiers in Psychology	China	Chinese (translated into English)	Video	Therapist: 3 full‐time therapists (1 female and 2 males) Client: 7 Chinese adolescents with depression (4 females and 3 males; aged 14–17) Type of therapy: not given	Qualitative
Voutilainen and Koivisto (2022)	Discourse Studies	Finland	Finnish (translated into English)	Video	Therapist: the trained psychoanalysts Client: clients Type of therapy: psychodynamic psychotherapy	Qualitative
Chourdaki et al. (2023)	Journal of Child Psychotherapy	UK	English	Audio	Therapist: psychotherapists Client: four adolescents with depression (two male, two female) Type of therapy: Short‐Term Psychoanalytic Psychotherapy	Qualitative
Muntigl et al. (2023)	Research on Language and Social Interaction	Canada	English	Audio, Video	Therapist: four female therapists Client: 7 clinical depression clients (five females, two males) Type of therapy: emotion‐focused therapy	Qualitative

### 2.5 Quality Evaluation

All the included studies adopted the CA method as the main research method. Given the unique epistemological orientation and analysis procedures, the mainstream quality assessment tools are not fully applicable to CA research. As CA studies rarely use the Joanna Briggs Institute (JBI) or CASP qualitative checklists for quality evaluation, we adopted the customized evaluation dimensions proposed by Parry and Land (2013) to assess each study in terms of both data type and analytical depth. We have systematically evaluated the multiple dimensions included in the research, including sample size, research design, and analysis procedures. Tables 3 and 4 provided detailed information.

**TABLE 3:** Quality appraisal of data.

Author(s) & Year	Overall size data collection	Number of episodes in collection(s)	Number of episodes from the collection that appear in the publication	Explicit reference to practices observed in more than one individual
Peräkylä (2005)	Not given	60 sessions	27 sessions	Yes
Voutilainen et al. (2010a)	Not given	57 sessions	26 sessions	Yes
Voutilainen et al. (2010b)	Not given	Not given	Not given	Yes
Voutilainen et al. (2011)	57 h	Not given	Not given	Yes
Muntigl et al. (2013)	15 h	Not given	15 sessions	Yes
Muntigl et al. (2014)	15 h	20 sessions	15 sessions	Yes
Muntigl and Horvath (2014a)	14 h	Not given	Not given	Yes
Muntigl and Horvath (2014b)	Not given	6 sessions	Not given	Yes
Sutherland et al. (2014)	Not given	Not given	8 sessions	Yes
Weiste and Peräkylä (2014)	60 h	221 sessions	15 sessions	Yes
Muntigl et al. (2019)	15 h	15 sessions	8 sessions	Yes
Muntigl (2020)	Not given	20 sessions	1 session	Yes
Deppermann et al. (2020)	Not given	50 sessions	Not given	Yes
Guxholli et al. (2021)	15 h	18 sessions	Not given	Yes
Ma et al. (2022)	15 h and 48 min	15 sessions	Not given	Yes
Voutilainen and Koivisto (2022)	Not given	30 sessions	12 sessions	Yes
Chourdaki et al. (2023)	Not given	35 sessions	10 sessions	Yes
Muntigl et al. (2023)	Not given	Not given	21 sessions	Yes

**TABLE 4:** Quality appraisal of analysis.

Author(s) and year	Examines more than one party's turns	Examines more than only topical content	Includes examination of aspects of the sequential environment	Includes examination of aspects of the turn	Includes examination of interactional effects	Includes examination of deviant cases	Are central analytic claims supported by direct quotes from or references to the data?	Reviewer's judgement of the degree to which the analysis is fine‐grained?	Are established analytic findings used as ‘tools’ in the analysis?
Peräkylä (2005)	Yes	Yes	Yes	Yes	Yes	No	Often	Very	Considerably
Voutilainen et al. (2010a)	Yes	Yes	Yes	Yes	Yes	No	Often	Very	Considerably
Voutilainen et al. (2010b)	Yes	Yes	Yes	Yes	Yes	No	Often	Very	Considerably
Voutilainen et al. (2011)	Yes	Yes	Yes	Yes	Yes	No	Often	Very	Considerably
Muntigl et al. (2013)	Yes	Yes	Yes	Yes	Yes	No	Often	Very	Considerably
Muntigl et al. (2014)	Yes	Yes	Yes	Yes	Yes	No	Often	Very	Considerably
Muntigl and Horvath (2014a)	Yes	Yes	Yes	Yes	Yes	No	Often	Very	Considerably
Muntigl and Horvath (2014b)	Yes	Yes	Yes	Yes	Yes	No	Often	Very	Considerably
Sutherland et al. (2014)	Yes	Yes	Yes	Yes	Yes	No	Often	Very	Considerably
Weiste and Peräkylä (2014)	Yes	Yes	Yes	Yes	Yes	No	Often	Very	Considerably
Muntigl et al. (2019)	Yes	Yes	Yes	Yes	Yes	No	Often	Very	Considerably
Deppermann et al. (2020)	Yes	Yes	Yes	Yes	Yes	No	Often	Very	Considerably
Muntigl (2020)	Yes	Yes	Yes	Yes	Yes	No	Often	Very	Considerably
Guxholli et al. (2021)	Yes	Yes	Yes	Yes	Yes	No	Often	Very	Considerably
Ma et al. (2022)	Yes	Yes	Yes	Yes	Yes	No	Often	Very	Considerably
Voutilainen and Koivisto (2022)	Yes	Yes	Yes	Yes	Yes	No	Often	Very	Considerably
Chourdaki et al. (2023)	Yes	Yes	Yes	Yes	Yes	No	Often	Very	Considerably
Muntigl et al. (2023)	Yes	Yes	Yes	Yes	Yes	No	Often	Very	Considerably

### 2.6 Data Synthesis

This study adopted a narrative comprehensive method to analyse and integrate the research results included in the literature. Narrative synthesis refers to a method of systematically reviewing and synthesizing multiple research results, mainly relying on words and texts to summarize and interpret the comprehensive results (Popay et al. 2006). We used thematic analysis to synthesize the research results, specifically adopting the six‐step process proposed by Braun and Clarke (2006): data familiarity, data coding, theme search, theme review, theme definition, and data analysis.

## 3 Results

After the screening process, a total of 18 studies meeting the inclusion criteria were included. The included studies were published between 2005 and 2023. These studies originated from six countries, with seven conducted in Canada, six in Finland, two in the UK, one in Germany, one in Albania, and one in China. The data primarily consisted of dyadic dialogues between the therapist and the client. Participants were mainly clients with emotional or affective disorders (e.g., depression, anxiety) and therapists, including the cognitive therapist, emotion‐focused therapist, client‐centred therapist, or psychoanalyst. The dataset consisted of 18 studies across five languages. Most studies were conducted in English (*n* = 12), whereas a smaller number involved Finnish (*n* = 3), Chinese (*n* = 1), German (*n* = 1), and Albanian (*n* = 1). For studies based on non‐English data, the original data were translated into English in the published extracts. All studies were qualitative and employed CA, focusing on psychotherapeutic settings. This review will present the existing body of research based on emotion talk in therapist‐client interaction within psychotherapeutic settings, which is organized around two main themes: sequential organization of emotion talk and changes in therapeutic interaction (see Table 5).

**TABLE 5:** Synthesized themes from included studies.

Themes	Subthemes	Theme descriptions
Sequential organization of emotion talk	Emotional displays	Client‐initiated emotional displays
		Therapist‐elicited emotional displays
	Management of clients' emotional displays	Affiliative responses
		Disaffiliative responses
	Responses to therapists' management	Alignment
		Resistance
		Ambivalence
Changes in therapeutic interaction	Changes in clients' experience	Changes in emotion
		Changes in referents
		Changes in cognition
		Changes in stances
	Changes in the therapeutic relationship	The disaffiliation of the relationship
		The re‐affiliation of the relationship

### 3.1 Sequential Organization of Emotion Talk

This theme focused on the sequential organization of emotion talk in psychotherapy. Specifically, it examined how clients displayed emotions, how therapists managed these emotional displays, and how clients responded to the therapists' management.

#### 3.1.1 Emotional Displays

The subtheme focused on how clients displayed emotions during the psychotherapy process. Across the reviewed studies, clients' emotional displays were shown to be accomplished through verbal and non‐verbal resources. In some cases, therapists also elicited such emotional displays (Muntigl 2020; Muntigl et al. 2023; Muntigl et al. 2019; Voutilainen et al. 2010b).

Clients' emotional displays were consistently described as relying on both verbal and non‐verbal resources, as well as varying in intensity (Muntigl et al. 2023; Muntigl et al. 2019; Voutilainen et al. 2010b). For instance, in cognitive‐constructivist psychotherapy, clients with depression employed intensive affective terms (e.g., ‘agonizing’, ‘anxious’) and extreme case formulations (e.g., ‘all the time’, ‘everything makes me stressed’) to intensify their expressions and convey inner suffering (Voutilainen et al. 2010b, 302). Based on data from the York I Depression Study, Muntigl et al. (2019) further demonstrated that clients drew on evaluative language, prosodic features, and facial expressions to construct self‐critical affective stances. These displays were associated with three interactional concerns: diminished control, reduced entitlements, and negative evaluations of the self. Furthermore, a reviewed study also revealed that clients' emotional displays in psychotherapy were organized along a clear intensity continuum (Muntigl et al. 2023). Drawing on data from the York I Depression Study, Muntigl et al. (2023) illustrated clusters of features associated with low‐intensity and high‐intensity distress displays. Low‐intensity emotional displays were characterized by pauses, audible breathing, sighs, lowered gaze, and shrugging, whereas high‐intensity distress was communicated through the co‐occurrence of multiple modalities, including sobbing, crying, facial expressions, shaking shoulders, lowered head, or burying the face in hands.

In addition to client‐initiated emotional displays, one study examined how therapists elicited clients' emotional displays. Based on data from the York I Depression Study, Muntigl (2020) examined how therapists guided clients' emotional work. Clients deferred their painful feelings by emphasizing vulnerability (e.g., I'm feeling tired, I'm a little delicate). Therapists responded by deploying immediacy questions and by carefully managing the timing and placement of their interventions. They additionally used imperative directives (e.g., so you let it out) or embodied actions, such as leaning physically towards the client, to facilitate clients' emotional displays. Through these practices, therapists guided clients from emotional avoidance towards overt emotional displays, for example, prompting them to break into intense crying that reveals their underlying distress.

#### 3.1.2 Management of Clients' Emotional Displays

One major line of research on the therapeutic alliance has examined how therapists respond to patients' emotional displays. The subtheme summarized the interactional practices that therapists employed to manage clients' emotional displays. Across the reviewed studies, therapists' practices were broadly categorized into affiliative and disaffiliative responses.

Nine studies collectively demonstrated how affiliative interactional practices, such as minimal responses, topic extension, noticing, immediacy questions, and formulations, can contribute to a supportive therapeutic environment and facilitate the therapeutic process. (Deppermann et al. 2020; Muntigl et al. 2023; Muntigl and Horvath 2014a; Muntigl et al. 2014; Muntigl et al. 2013; Sutherland et al. 2014; Voutilainen and Koivisto 2022; Voutilainen et al. 2010a; Weiste and Peräkylä 2014). Specifically, therapists confirmed or acknowledged clients' emotions, encouraged further emotional displays, and interpreted these emotions to promote self‐reflection. The reviewed studies showed that therapists extended topics through utterances and intonation to acknowledge the client's stance (Voutilainen et al. 2010a; Weiste and Peräkylä 2014). In a cognitive‐constructivist psychotherapy study conducted in Finland, therapists employed an and‐prefaced turn to frame their utterance as an extension of the client's narrative, for example, ‘And there they were now again, all of them together’ (Voutilainen et al. 2010a, 91). Weiste and Peräkylä (2014) demonstrated that therapists could extend the intonation of the client's prior turn by using a lower or quieter voice, maintaining prosodic continuity with the client while also creating a validating trajectory. Additionally, minimal responses were employed to convey therapists' understanding of clients' emotions. Evidence from the two‐chair self‐soothing task in Emotion‐Focused Therapy (EFT) demonstrated that therapists employed response tokens to maintain interactional continuity and convey emotional support, such as ‘hmmm’, ‘yeah’, and ‘good’ (Sutherland et al. 2014, 747). Simultaneously, therapists employed noticing strategies or immediacy questions to further guide clients in displaying more emotions, facilitating emotion talk (Muntigl et al. 2023; Muntigl and Horvath 2014a). Therapists employed noticing strategies to encourage clients to elaborate on their emotional experiences, maintaining focus on emotional topics, and facilitating more emotion talk, for example, by using prompts such as ‘tell me about whichever one is most strong for you’ or “I can see some sadness in your eyes” (Muntigl and Horvath 2014a, 95). By drawing on the data from the York I Depression Study, Muntigl et al. (2023) showed that, in addition to using noticing strategies, therapists also employed immediacy questions to guide clients in attending to and reflecting on their immediate emotional experiences (e.g., ‘Something is happening there? You're sighing?’ or ‘What I'm saying is making you teary, right?’, pp. 17–19). Furthermore, therapists were also described as engaging in interpretative practices that formulated clients' emotional experiences (Deppermann et al. 2020; Muntigl et al. 2014). Therapists attended to clients' verbal and non‐verbal conduct and provided an interpretation that assigns explicit emotional interpretations, such as ‘it makes you kinda little bit sad, though this thought’ (Deppermann et al. 2020, 12). Similarly, drawing on the video data from the York Depression Study, Muntigl et al. (2014) identified three practices through which therapists responded to clients' emotional displays: eliciting, naming, and illustrating emotional impact. Among these practices, illustrating constituted the strongest affiliative response. Illustrating relied on multimodal resources, including graphic language, metaphorical expressions, and embodied actions, to render emotional experiences more vivid.

In contrast, delayed responses represented a more nuanced form of empathy in therapeutic interactions, leaving clients space to elaborate on their emotional experience (Muntigl et al. 2013; Voutilainen and Koivisto 2022). In psychodynamic psychotherapy, therapists employed silence when confronted with patients' negative emotions, allowing patients space to engage in self‐explanation. Therapists subsequently responded with contrastive comments (e.g., ‘yeah but’) or candidate understandings (e.g., ‘so that’), achieving empathy through temporal delay (Voutilainen and Koivisto 2022, 253–259). Similarly, in EFT, nonverbal delayed responses served a comparable affiliative function. Therapists initially refrained from immediately nodding in response to clients' disaffiliative stances, instead delaying the nod until the client had fully articulated their emotional stance, at which point the nod aligned with the expressed emotion (Muntigl et al. 2013). Through precise timing, this nonverbal cue signalled active retreating while simultaneously establishing affiliation with the client. This approach avoided premature intervention and provided space for emotions to unfold naturally.

Across four studies, a second pattern of disaffiliative practices was identified in which therapists ignored or even challenged patients' emotional displays (Chourdaki et al. 2023; Muntigl and Horvath 2014a; Voutilainen et al. 2010b; Weiste and Peräkylä 2014). One recurrent practice was topical or temporal shifting, which reoriented the interaction away from the client's emotional displays. In a UK study on Short‐Term Psychoanalytic Psychotherapy (STPP) for adolescent depression, when adolescents displayed anger towards the therapist, the therapist employed formulations such as ‘we are not talking about’ and ‘that's today’ to implement topical or temporal shifts, thereby creating distance from their emotions (Chourdaki et al. 2023, 286–288). Another disaffiliative response involved directly challenging clients' emotional displays, prompting them to re‐examine their narratives or feelings (Muntigl and Horvath 2014a; Voutilainen et al. 2010a; Weiste and Peräkylä 2014). One way of challenging clients' emotions was through prosodic disjuncture (Weiste and Peräkylä 2014). When therapists offered formulations, their prosodic features sometimes did not align with the client's speech rhythm, manifesting as increased pitch, greater loudness, and sudden intonational shifts (Weiste and Peräkylä 2014). Similarly, therapists employed noticing to point out contradictions between the patient's verbal and nonverbal behaviours and question the patient's emotions (Muntigl and Horvath 2014a). The therapist noticed the inconsistency between the patient's words and behaviours and then employed ‘I don't understand’ to express their own confusion, such as ‘You say you're scared, but you're smiling. I don't understand how one can be scared and smile at the same time’ (Muntigl and Horvath 2014a, 102). Additionally, therapists guided clients to re‐examine their own feelings through questions or requests for confirmation (Voutilainen et al. 2010b). As in ‘So you are saying that you feel at home for real’, where the emphasis on ‘real’ conveyed a sceptical stance (Voutilainen et al. 2010b, 305).

#### 3.1.3 Responses to Therapists' Management

This subtheme explicated clients' responses to therapists' management of their emotional displays. Across the reviewed studies, three types of responses therapists' management were identified: alignment, resistance, and ambivalence (Chourdaki et al. 2023; Muntigl 2020; Muntigl and Horvath 2014a; Muntigl et al. 2014; Muntigl et al. 2013; Peräkylä 2005; Voutilainen et al. 2011).

Clients displayed alignment with therapists' emotional management through various practices (Peräkylä 2005; Voutilainen et al. 2011). One basic form of such alignment involved the use of acknowledgement tokens (Voutilainen et al. 2011). The client responded to the conclusive interpretation of the therapist with utterances like ‘yeah’ and ‘that's the way it is’, confirming the therapist's understanding and demonstrating alignment and consensus (Voutilainen et al. 2011, 360). Beyond these minimal acknowledgements, patients further employed extended elaboration to continue to extend the therapist's perspective, displaying a deeper level of alignment (Peräkylä 2005). These elaborations of the interpretation showed the patient's agreement with the therapist's interpretation and represented an active co‐construction of the therapeutic collaboration (Peräkylä 2005).

Across studies, resistance was shown to be implemented through a range of interactional practices that withhold or challenge the therapist's emotional exploration (Chourdaki et al. 2023; Muntigl 2020; Muntigl and Horvath 2014a; Muntigl et al. 2013; Voutilainen et al. 2011). One recurrent form involved delayed responses, which functioned to withhold uptake of the therapist's emotional invitation and to resist emotional exploration (Muntigl and Horvath 2014a; Voutilainen et al. 2011). Based on the data of psychotherapy treatments with an experiential focus in Canada, Muntigl and Horvath (2014a) demonstrated that patients used silence to express hesitation and reluctance, thus rejecting the therapist's guiding intervention. Clients also employed hesitant or ambiguous expressions, such as ‘It didn't necessarily mean that… but I do feel…’, to convey a subtle form of resistance (Voutilainen et al. 2011, 353). In addition, one response drawing on vulnerability displays could function to resist emotional exploration. Muntigl (2020) pointed out that clients also emphasized their vulnerability to resist the therapist's efforts, such as using words such as ‘not having good resources’, ‘being tired’, or ‘feeling delicate’ to create a fragile and helpless image (p. 7). The reviewed studies showed that the client adopted explicit resistance to maintain his position (Chourdaki et al. 2023; Muntigl 2020; Muntigl et al. 2013). When a therapist's formulation was perceived by the patient as not matching their own experience, it was treated as a challenge to their epistemic rights, resulting in disagreements (Muntigl et al. 2013). Therefore, clients employed various forms to resist the therapist's interventions, such as sarcasm, criticism, or antagonistic expression (Chourdaki et al. 2023; Muntigl 2020). one study demonstrated that clients articulated explicit resistance by using sarcasm or criticism, such as ‘gee, when you put it like that’, ‘I don't know’, and ‘I never have’ (Muntigl 2020, 10). This phenomenon was particularly prominent in adolescent psychotherapy, as proven by the repeated occurrence of such behaviours in adolescents (Chourdaki et al. 2023). In the UK, Chourdaki et al. (2023) revealed that adolescent clients with depression resisted through antagonistic emotional expression, including interrupting (‘but’, ‘I just don't’), raising their tone, and using sarcasm and swear words (e.g., ‘shit’, ‘stupid’, ‘crap’) to defend their own stance (pp. 287–292).

Compared to alignment or resistance, a third type of response identified across studies was ambiguous or low‐intensity acceptance (Muntigl et al. 2014; Voutilainen et al. 2011). Specifically, patients initially agreed with the therapist's conclusion, then used qualifiers (‘perhaps’) or self‐reflective talk (e.g., ‘I still don't want to’, ‘I don't know’) to readjust their stance, displaying an emotional state of ‘not yet ready to accept’ (Voutilainen et al. 2011, 354–355). Similarly, in a Canadian study on EFT, Muntigl et al. (2014) presented that although clients responded with emphatic expressions like ey(h)eah and nodding, indicating overt agreement, they simultaneously resisted the therapist's attempt to focus the narrative by shifting the subject of shared experiences.

### 3.2 Changes in Therapeutic Interaction

The psychotherapy process involves the transformation of experience (Peräkylä 2019). The theme discussed changes in therapeutic interaction, particularly focusing on changes in clients' experience and changes in the therapeutic relationship.

#### 3.2.1 Changes in Clients' Experience

This subtheme delineated how therapists used interactional practices to facilitate changes in clients' experiences. Across four studies, changes were reported in four facets: emotion, referents, cognition, and stances (Ma et al. 2022; Muntigl et al. 2023; Sutherland et al. 2014; Voutilainen et al. 2011).

Across studies, cognitive change was commonly realized through interactional practices that reconstructed clients' understanding of their emotions. In their study on transformative sequences conducted in the Chinese psychological counselling clinic, Ma et al. (2022) demonstrated that therapists guided adolescents with depression through practices such as formulation and interpretation, transforming cognition during psychotherapy, from a state of unknowing or unawareness of their emotional issues towards gradually becoming aware of them. Therapists facilitated shifts in referents to reframe clients' focus and maintain the interactional relationship (Ma et al. 2022; Sutherland et al. 2014). In a study on EFT conducted in the UK, Sutherland et al. (2014) revealed that therapists deliberately guided clients through the shift of person reference, particularly from the third person to the second person, facilitating movement and connection between different self‐positions. Therapists also adopted interpretive formulations to discuss and transform referents, shifting the client's focus from external behaviours to internal thoughts (Ma et al. 2022).

The reviewed studies showed that therapists employed various interactional practices to regulate clients' emotions, either intensifying or attenuating their emotional experiences to promote emotional changes (Ma et al. 2022; Muntigl et al. 2023). Lexical substitution was one of the most common methods of emotion regulation (Ma et al. 2022). Therapists attenuated clients' negative emotions by replacing lexical terms, for example, substituting “much” with “little”, “very bad” with “a little bad” (Ma et al. 2022, 10). This approach conveyed understanding while gradually reducing the intensity of the negative emotions described by clients, thereby reframing their perception of problem severity (Ma et al. 2022). In addition, therapists also employed modulating directives to transform clients' emotions (Muntigl et al. 2023). Specifically, they employed directives that either sustained or reduced emotional intensity as a means of regulating the client's current emotional state. To sustain emotional experience, they used directives such as ‘stay there’ or ‘stay with it’ (Muntigl et al. 2023, 27). When emotions became overly intense, they guided the client to calm down with prompts like “take a breath” (Muntigl et al. 2023, 32).

A study found that clients' stances were progressively recast, reflecting shifts between therapists' conclusions and clients' responses (Voutilainen et al. 2011). In Finland, Voutilainen et al. (2011) identified three stages in the transformation of clients' responses in cognitive‐constructivist psychotherapy. In the initial stage, clients displayed resistance through prolonged silences or indirect expressions. During the transitional phase, while beginning to acknowledge the therapist's perspective, clients maintained psychological distance using qualifiers. By the later stage, clients gradually accepted and internalized the therapist's reinterpretations, expressing agreement and resonance through repeated phrases such as ‘that's the way it is,’ ‘yeah,’ and ‘Mmmm’ (Voutilainen et al. 2011, 360).

#### 3.2.2 Changes in the Therapeutic Relationship

The subtheme focused on changes in the therapeutic relationship. Six studies elaborated processes of disaffiliation and re‐affiliation within the alliance (Guxholli et al. 2021; Ma et al. 2022; Muntigl 2020; Muntigl and Horvath 2014b; Muntigl et al. 2013; Voutilainen et al. 2010b).

Across three studies, it was shown that the disaffiliation in the therapeutic relationship stemmed from client negativity, rejection of client empathy‐seeking, and inappropriate repair practices (Muntigl 2020; Muntigl et al. 2013; Voutilainen et al. 2010b). Therapists' formulations in response to clients' distress could occasion clients' criticism or reproach towards the therapist. These negative affective responses could escalate to the relational level, causing an alliance rupture (Muntigl 2020). Similarly, drawing on the data of cognitive‐constructivist psychotherapy in Finland, Voutilainen et al. (2010b) showed that therapists' rejection of clients' attempts to seek empathy could intensify tension in the interaction and lead to a rupture in the therapeutic alliance. Furthermore, the use of inappropriate repair practices could further exacerbate the disaffiliation and divergence of the relationship (Muntigl et al. 2013). Therapists engaged in repair practices that maintained incongruent perspectives vis‐à‐vis the client, deploying contrastive markers such as ‘no I mean, I thought that's what you were saying…’ or ‘yeah of course of course. but…’ (Muntigl et al. 2013, 15). Rather than re‐establishing affiliation, these practices intensified the disagreement. A study on the therapeutic relationship in EFT in Canada noted that clients' manners of disaffiliation were categorized into two types: practices of confrontation and practices of withdrawal (Muntigl and Horvath 2014b). Confrontation occurred when the therapist and the client worked towards different agendas. The client obstructed the therapist's agenda through direct disagreement, extended self‐advocacy, and interruptions, which left the therapist unable to express empathy. Clients' practices of withdrawal arose when clients employed various evasion strategies to avoid engaging with the therapist's formulations (Muntigl and Horvath 2014b).

Other studies highlighted that therapists also employed interactional practices to address disaffiliation and re‐affiliation (Guxholli et al. 2021; Muntigl 2020; Muntigl et al. 2013; Voutilainen et al. 2010b). Collaborative moves were shown to display that the therapist was collaboratively following the patient's account, with practices including collaborative completions, formulations, and extensions (Guxholli et al. 2021). However, such moves were primarily oriented towards managing or alleviating disaffiliation rather than achieving full re‐affiliation with the patient. Two studies noted that a further way of addressing alliance ruptures was to explore relational themes (Muntigl 2020; Voutilainen et al. 2010b). Therapists responded by attending to clients' emotions towards the therapist (Muntigl 2020). Shifting the focus to the here‐and‐now relational context enabled therapists to guide both parties in collaboratively examining the emergent dynamics of their interaction, thereby working to resolve tensions. For example, therapists used expressions such as ‘do you feel that something might come from me’, ‘I began to think’, or ‘I thought’ to invite clients to reflect on the ongoing therapeutic relationship (Voutilainen et al. 2010b, 308–310). In addition, the re‐affiliation of the therapeutic relationship also relied on specific repair devices (Muntigl et al. 2013). Muntigl et al. (2013) identified a form of repair device that assumed congruency of perspectives: therapists employed affiliating devices such as nods and repair initiators (e.g., ‘that's what I meant…’, ‘yeah, yeah, I'm saying that yeah…’) to display alignment with the client's stances, thereby maintaining an affiliative relationship (p. 10).

In addition to therapist‐initiated practices, re‐affiliation also emerged as a joint achievement between therapist and client. Muntigl and Horvath (2014b) pointed out that the client engaged in the exploration of their personal concerns, personal problems in depth, whereas the therapist drew on a range of interactional resources to affiliate with the client. Following extended silence, clients could initiate new topics (e.g., ‘in touch with her emotions’, p. 339) as a form of strategic concession. In turn, the therapist used positive appraisals and upgraded confirmation to respond (e.g., ‘this is wonderful’, ‘mm hm! Mm hm’, p. 339), thereby reinforcing affiliation.

## 4 Discussion

This review synthesized 18 CA studies on emotion talk in psychotherapy and identified two main themes: (1) sequential organization of emotion talk, focusing on emotional displays, emotional management, and responses to therapists' management; and (2) changes in therapeutic interaction, including changes in clients' experience and changes in the therapeutic relationship. To the best of our knowledge, this review is the first systematic synthesis of CA studies specifically focused on emotion talk in psychotherapy. By integrating and extending prior strands of research, this review helps to reveal the micro‐mechanism of emotion talk, clarifying how emotion is generated, responded to, and managed in therapeutic interaction. It synthesizes the interactional practices through which clients display emotion, therapists respond to and manage these displays, and participants deal with moments of disaffiliation, offering practice‐relevant insights into how therapists may attend to, respond to, and work with clients' emotional displays in interaction.

Across the reviewed studies, clients' emotional displays are consistently treated as interactional resources that shape the trajectory of therapeutic sequences. Taken together, these studies show that clients display emotions through verbal and non‐verbal resources, including affective terms, sobbing, crying, or facial expressions (Voutilainen et al. 2010b; Muntigl et al. 2019). The synthesis of these studies clarifies that clients' emotional displays bring participants closer to the core of the psychotherapeutic activity and occasion or mobilize a next action, providing opportunities for further therapeutic work. By attending to the intensity of the emotional display, therapists may manage clients' emotions through different response practices, such as affiliation, elaboration, interpretation, or topical shift (Muntigl et al. 2023). As documented in the broader CA studies, emotional displays can function as weak invitations or as practices that mobilize a response from co‐participants (Stivers and Rossano 2010). In therapeutic contexts specifically, clients may actively seek empathic responses from therapists through behaviors such as wet sniffs (Hepburn and Potter 2007). Furthermore, the present review integrates multiple interactional resources through which clients display emotions, highlighting their role in organizing therapeutic sequences and in mobilizing specific forms of therapist response.

Synthesizing these individual studies, a second contribution of this review is to identify two categories of therapist response: affiliative and disaffiliative responses. Therapists employ various interactional practices (e.g., minimal tokens, extended topics, noticing) to manage emotion through affiliative responses, which confirm or acknowledge clients' emotional experiences and further facilitate emotional exploration (Muntigl and Horvath 2014a; Muntigl et al. 2023; Sutherland et al. 2014; Voutilainen et al. 2010a). In contrast, disaffiliative responses tend to involve less emotional exploration or challenge clients' emotions through various practices, such as shifting topic, prosodic disjuncture and requests for confirmation (Chourdaki et al. 2023; Voutilainen et al. 2010a; Weiste and Peräkylä 2014). The synthesis indicates that the difference between affiliative and disaffiliative responses is not only a type difference, but has practical consequences: affiliative responses tend to support the continuation of emotional exploration and the maintenance of affiliation, whereas disaffiliative responses may be followed by reduced emotional elaboration and interactional difficulties, thereby rendering relational repair relevant. Consistent with broader research, affiliative responses can establish communicative attunement and empathetic moments, thus strengthening the therapeutic relationship by showing mutual understanding and empathetic responses (Elliott et al. 2011; Heritage 2011). On the contrary, certain behaviours of therapists during treatment, such as passive attitudes and minimal responses, may cause clients' dissatisfaction and anger (O' Keeffe et al. 2020). The reviewed studies therefore suggest that affiliative responses are one important resource through which therapists can support emotional exploration and maintain the therapeutic relationship.

The present review also underlines how therapeutic changes occur in emotion talk. In the broader CA studies in psychotherapy, therapeutic change has been described in terms of transformations in clients' experience, including changes in emotion, reference, and relation, and clarified how the sequence structure in psychotherapy promotes the transformation of clients' experience through interactional practices (Bercelli et al. 2013; Peräkylä 2019). In the studies included in this review, emotion talk has proven to be an important place where such transformations may be initiated in interactions. Importantly, the additional dimension of cognitive transformation is proposed in one of the reviewed studies (Ma et al. 2022). It enables clients to identify and confront their own emotional problems, thus laying the foundation for subsequent treatment (Ma et al. 2022). Therefore, this review explains how clients' experiences are managed and changed in psychotherapy, offering empirically grounded insights into how therapeutic work may be advanced through the moment‐by‐moment management of emotion talk.

The synthesis further highlights the importance of the therapeutic relationship in emotion talk. The current studies show that the clients' anger and aggressive behaviour may adversely affect the therapeutic relationship and contribute to treatment discontinuation (Halfon et al. 2019; Topel and Lachmann 2007). In the research reviewed, the therapist's practices for addressing disaffiliation, restoring alignment and re‐establishing engagement emerge as a concern in the interaction, including assumed congruency of perspectives, collaborative moves and exploration of relational themes, serving to support the ongoing therapeutic relationship and create conditions for further therapeutic work (Guxholli et al. 2021; Muntigl 2020; Muntigl et al. 2013; Voutilainen et al. 2010b). By integrating these findings, the present review clarifies that emotion talk is related to the ongoing maintenance and repair of the therapeutic relationship.

The review synthesizes the interactional resources of emotion talk in psychotherapeutic settings, offering valuable insights into the processes and effectiveness of psychotherapy. First, the synthesis highlights the importance of clients' emotional displays and integrates how they function as interactional resources. Therapists should thus attend to and guide clients' emotional expressions; for example, they may encourage emotional displays by using questions to prompt further emotional expression (Muntigl 2020). Second, this review reports how the therapist's management of clients' emotional displays shapes the course of psychotherapy. Affiliative responses, in particular, facilitate further exploration of clients' emotions and promote continued emotion talk. Accordingly, therapists should benefit from employing interactional practices to establish empathic relationships with clients, such as formulations, empathic responses and interpretative moves, which display understanding and recognition of clients' emotional experiences (Muntigl et al. 2014; Muntigl et al. 2023; Weiste and Peräkylä 2014). Third, therapeutic change constitutes a central component of the therapeutic process, as one of the central tasks of psychotherapy is treating clients' emotional disorders and promoting changes in their emotions and behaviours (Antaki 2008; Peräkylä et al. 2008b; Peräkylä 2019). Therapists should employ a range of interactional practices, such as formulations, lexical substitution, and interpretations, which support changes in clients' experiences and the interactional relationship (Ma et al. 2022).

Furthermore, using Parry and Land's (2013) customized dimensions for CA research, we assessed the included studies in terms of data type and analytical depth. Table 3 summarizes data‐related features, including data scale and discourse fragment distribution. Table 4 examines sequence organization and analytic grounding. The appraisal suggests that the included studies generally meet core CA standards, particularly in their use of naturally occurring data, sequential analysis and close grounding of claims in interactional evidence. The quality appraisal informs how the synthesis should be interpreted. Because the included studies are generally grounded in naturally occurring data and detailed sequential analysis, the review can credibly synthesize recurrent interactional practices through which emotion talk is displayed, responded to, and managed. However, because several studies draw on small or modality‐specific corpora, and because deviant cases are not always explicitly discussed, the synthesis should not be read as establishing the prevalence, universality, or outcome effectiveness of particular practices across psychotherapy as a whole. Instead, it identifies interactional resources documented in existing CA research and clarifies the sequential environments in which they have been shown to operate.

This review adheres to standard systematic review procedures. However, several limitations should be acknowledged. First, we did not register our review on PROSPERO or OSF. To enhance transparency and facilitate comparability, future studies should be registered on PROSPERO or OSF, which allows for pre‐specification of methods, reduces reporting bias and strengthens the credibility of the findings. Second, we only included the studies published in Chinese and English, which may have resulted in the exclusion of high‐quality studies published in other languages. Third, the inclusion criteria were limited to journals indexed in SCI and SSCI databases, potentially leading to the omission of relevant research published in non‐indexed journals, conference proceedings, or other scholarly venues. These limitations may somewhat compromise the comprehensiveness and generalizability of the findings. Future reviews should thus incorporate literature in more languages and extend to various publication types to achieve a more comprehensive evidence base.

## Author Contributions

**Shuai Zhang:** conceptualization, project administration, formal analysis, writing – original draft, supervision, writing – review and editing. **Tiantian Huang:** conceptualization, investigation, formal analysis, writing – original draft, writing – review and editing. **Haiying Li:** investigation, project administration, supervision, writing – review and editing. **Lu Chen:** methodology, formal analysis, investigation, project administration, supervision, writing – review and editing.

## Funding

This work was supported by the Humanities and Social Sciences Youth Foundation, Ministry of Education (10.13039/501100017630, 22YJC740100), Shandong Provincial Social Science Planning General Project (25CYYJ02), Humanities and Social Sciences Project at University of Jinan (XR2502) and the National Social Science Fund of China General Project (25BYY066).

## Ethics Statement

This article does not contain any studies with human participants performed by any of the authors.

## Consent

This article does not contain any studies with human participants performed by any of the authors.

## Conflicts of Interest

The authors declare no conflicts of interest.

## Data Availability

No datasets were generated or analyzed during this study. All data reviewed are derived from previously published studies.
